# The Human Ear

**Published:** 1882-04

**Authors:** 


THE HUMAN EAR AND ITS DISEASES: A Practical Treatise upon the Examination, Recognition and Treatment of Affections of the Ear and Associate Parts, etc. By *W. H. Winslow, M. D., Ph. D.* Pp. 526. New York and Phila.: Boericke & Tafel. 1882.

Dr. Winslow gives, in this work, a treatise on Anatomy, Physiology and Diseases of the Ear, which differs slightly, if at all, from such works published by allopathic authors.

